# Effect of Vibrations, Displacement, Pressure, Temperature and Humidity on the Resistance and Impedance of the Shockproof Resistors Based on Rubber and Jelly (NiPc–CNT–Oil) Composites

**DOI:** 10.3390/gels8040226

**Published:** 2022-04-07

**Authors:** Muhammad Tariq Saeed Chani, Khasan S. Karimov, Abdullah M. Asiri, Mohammed M. Rahman, Tahseen Kamal

**Affiliations:** 1 Center of Excellence for Advanced Materials Research, King Abdulaziz University, P.O. Box 80203, Jeddah 21589, Saudi Arabia; aasiri2@kau.edu.sa (A.M.A.); mmrahman@kau.edu.sa (M.M.R.); tkkhan@kau.edu.sa (T.K.); 2 Chemistry Department, Faculty of Science, King Abdulaziz University, P.O. Box 80203, Jeddah 21589, Saudi Arabia; 3 Ghulam Ishaq Khan Institute of Engineering Sciences and Technology, Topi 23640, Pakistan; khasan@giki.edu.pk; 4 Center for Innovative Development of Science and Technologies of Academy of Sciences, Rudaki Ave., 33, Dushanbe 734025, Tajikistan

**Keywords:** resistor, organic semiconductor, nickel phthalocyanine, edible oil, flexible devices

## Abstract

Here, we present the design, fabrication and characterization of shockproof rubber–jelly (NiPc–CNT–oil) composite-based resistors. To fabricate the resistors, gels of CNT and NiPc with edible oil were prepared and deposited on a flexible rubber substrate using rubbing-in technique. The devices’ resistance and impedance were investigated under the effect of pressure, displacement, humidity, temperature and mechanical vibrations. The resistance and the impedance decreased, on average, by 1.08 times under the effect of pressure (up to 850 gf/cm^2^) and by 1.04 times under the effect of displacement (up to 50 µm). Accordingly, upon increasing the humidity from 60% to 90% RH, the resistance and impedance decreased by up to 1.04 times, while upon increasing the temperature from 25 °C to 43 °C, the resistance and impedances also decreased by up to 1.05 times. Moreover, under the effect of vibration, a decrease in resistance and impedance, by up to 1.03 times, was observed. The investigated samples can potentially be used as prototypes for the development of shockproof jelly electronic-based devices in particular resistors. The technological achievement in the fabrication of these devices is the use of edible organic oil, which allows for the fabrication of uniform jelly films of organic materials that cannot be realized simply by mixing “dry” ingredients. Especially, we highlight that edible organic oil is environmentally friendly, unlike some other inorganic oils that are used in practice.

## 1. Introduction

During the last few years, organic materials-based electronic devices have been investigated very intensively, especially in the area of sensor fabrication and investigation. On the other hand, a number of organic materials-based devices, particularly those with resistive properties, were also recently fabricated [1,2,3,4,5,6,7,8,9]. The investigation of PEDOT:PSS-based printed (inkjetted) resistors (organic) was described in ref. [1]. This resistor showed a small change in resistance with the change in temperature or humidity. When changing the temperature from 42 °C to 120 °C, the resistance changed from 17 kΩ to 15.6 kΩ. Similarly, when changing the humidity from 30 to 85 % RH, the resistance decreased from 15.4 to 15.0 kΩ. The electrohydrodynamic printing of organic polymeric resistors on the flat and uneven surfaces was presented in ref. [2]. Polymer-based resistive fully printed stable read-only memory and its application in mobile read out systems was described in ref. [3]. The fabrication of low-pass RC filters resistors through self-aligned inkjet printing on roll-to-roll imprinted plastics was presented in ref. [4]. The resistances of these resistors ranged from 10 to 106 Ω. A patented carbon-composition resistor was described in ref. [5], and the process of producing an electrical resistor was explained in ref. [6]. The resistance (electrical) element was comprised of metal resinate and powdered glass, where the metal resinate was admixed with precious metal powder using an organic vehicle (ethyl cellulose dissolved in alcohol). The resistance of the element was measured as 200 kΩ/square, and the resistor composition was described in ref. [7]. The resistors consisted of particles of refractory metal carbides (SiC), oxy-carbides and nonreducing-glass dispersed in organic vehicles. The resistors of 26.2 kΩ to 8.4 MΩ were fabricated by varying the composition. The patented polytetrafluoro ethylene lubricant for carbon-based resistors was presented in ref. [8]. A high-temperature resistor consisting of a sintered, uniformly dispersed mixture of a poly halo ethylene resin, which contained fluorine atoms in ethylene units with electrically conducting and electrically non-conducting particles, was presented in ref. [9].

Details of the fabrication, investigations and properties of various types of resistors were also provided in the refs. [10,11,12,13,14,15]. The organic resistors with positive characteristics were described in ref. [10]. The composition and process of producing electrical resistors were presented in refs. [11,12]. The method of manufacturing resistor paste was presented in ref. [13], while the composition and the properties of polymer thick-film resistors [14] and non-lead resistors [15] were also described.

In recent years, a number of papers related to resistive/impedimetric sensors were published by us [16,17,18,19,20,21,22,23,24,25,26,27,28]. In ref. [16], CNTs and graphene-based multifunctional sensors were fabricated and investigated for displacement, pressure and temperature-gradient sensing. The graphene and orange dye solid-electrolyte-cell-based humidity sensors were also studied [17]. The nanocomposites of chitosan-CuMn_2_O_4_ spinel were also investigated for impedimetric temperature and humidity sensing [18]. In ref. [29], a nickel-phthalocyanine-based photo field effect transistor was fabricated and investigated for the humidity sensing.

In a continuation of our efforts to study organic semiconductor and conductor devices, we present data on the fabrication, properties and investigation of shockproof jelly (NiPc–CNT–oil)–rubber composite-based resistors. The effect of pressure, displacement, humidity, temperature and vibrations on the performance of resistors has previously been studied. For the fabrication of organic-materials-based resistors, a unique combination of materials—edible oil, nickel phthalocyanine, carbon nanotubes and rubber (substrate)—was used. A technological achievement in the fabrication of these resistors is the use of edible organic oils, which allow the uniform jelly films of organic materials, which could not be realized simply by mixing “dry” ingredients, to be fabricated. These fabricated, flexible resistors provide an approximately constant resistance. The used organic materials and fabrication technology (rubbing-in technology) make the resistor ecologically clean. This device is ecologically clean not only from the point of fabrication, but also for practical utilization. Moreover, the fabricated resistors may potentially be used as a prototype for the development of shockproof-jelly-based electronic devices, particularly resistors.

## 2. Results and Discussion

**Figure 1:**
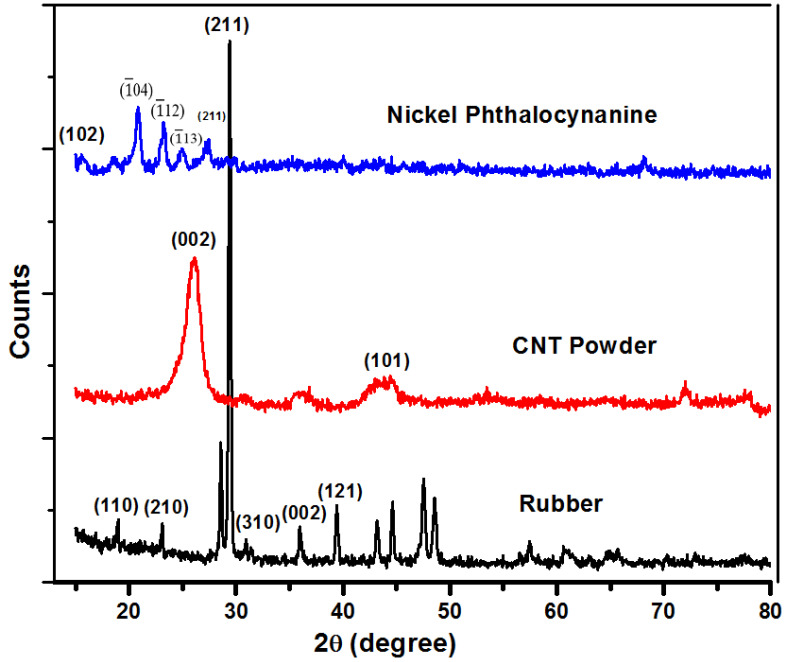
XRD patterns of original rubber, CNTs and nickel phthalocyanine powders.

Figure 1 shows the XRD spectra of the NiPc, CNTs and rubber substrate. The Philips PW1830 X-ray system was used in θ–2θ (Bragg–Brentano) scan mode using Cu-Kα radiation (monochromatic) with 40 kV (accelerating voltage) and 25 mA (tube current) at room temperature. The scanning was conducted in a 2θ range from 15° to 80°, while the step size was 0.05°. The peaks shown in NiPc spectrum are consistent with previously reported results [30,31]. The high-intensity peaks in the XRD pattern of rubber show the presence of polyvinylchloride and are in agreement with previous studies [32]. Similarly, the peaks of CNT also matched with previously reported patterns [33]. To confirm repeatability, the samples of rubber, CNTs and NiPc were scanned three times.

**Figure 2:**
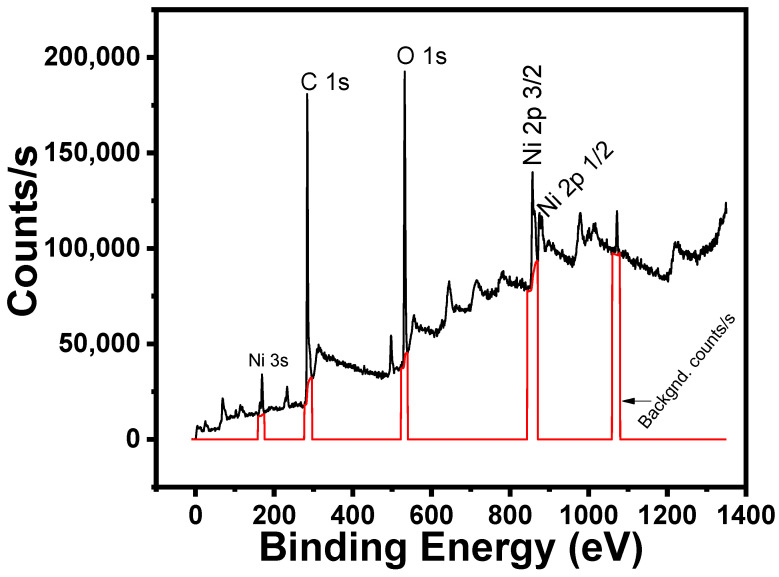
XPS spectrum of the CNT and NiPc composite showing the presence of Ni, C and O.

The XPS spectrum of the CNT-NiPc composite is shown in Figure 2. The analysis was carried out by using K-Alpha spectrometer with exciting source (radiation): Al K-α; spot size: 400 µm (beam); pass energy: 200.0 eV; step size: 1.0 eV (energy); and acquisition time: 1.0 min 8 s. The results shown in Figure 2 are compatible with the results presented in previous studies [31,34,35].

The samples’ resistance and impedance were investigated under the effect of displacement, pressure, humidity, temperature and mechanical vibrations. The resistance and the impedance of the surface type samples were equal to 2.2 kΩ, on average.

**Figure 3:**
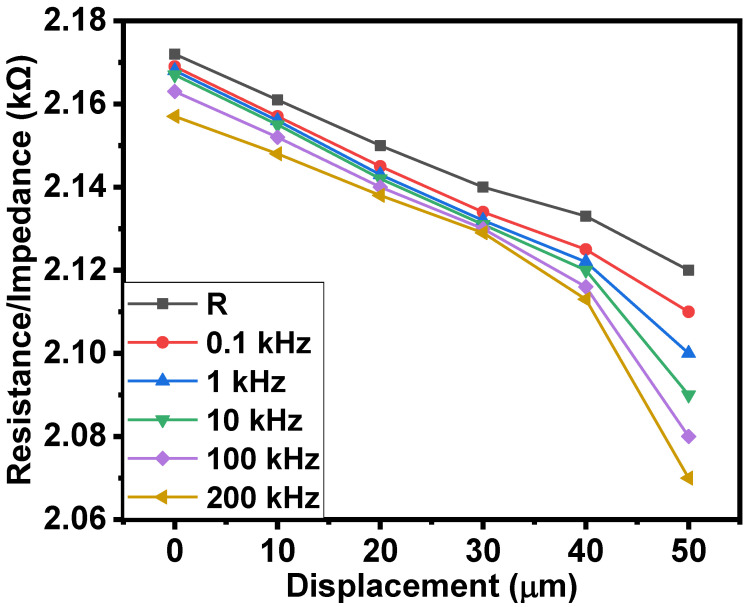
Resistance/impedance–compressive displacement relationships of the surface-type shockproof resistor based on NiPc–oil jelly and CNT–oil jelly rubber composite.

**Figure 4:**
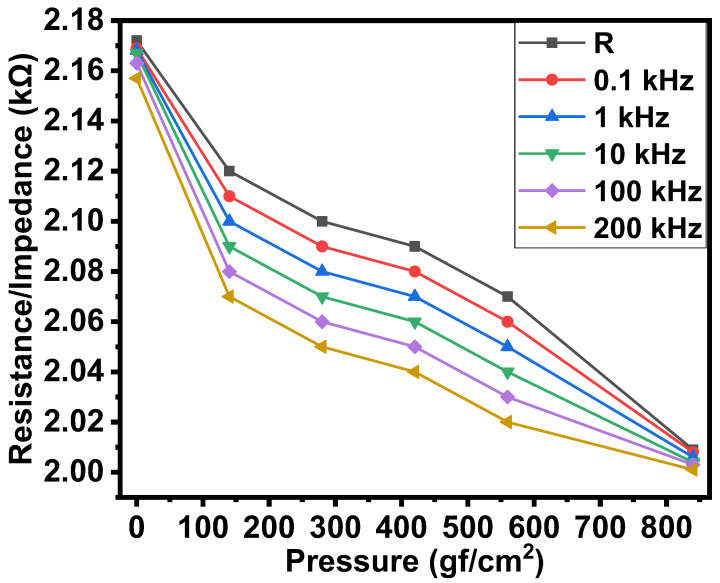
Resistance/impedances-pressure relationships of the surface type shockproof resistor based on NiPc–oil jelly and CNT–oil jelly rubber composite.

Figure 3 shows the dependence of the resistance and impedance (at various frequencies) of the surface-type, shockproof, NiPc-edible oil jelly and CNTs-edible oil–rubber composite-based resistors on compressive displacement. It could be seen that, as the compressive displacement increased from 0 to 50 µm, the resistance and impedances decreased by 1.04 times on average. Under the effect of pressure from 0 to 850 gf/cm^2^, the resistance and impedance decreased by 1.08 times on average. The results of resistance/impedance–pressure relationships are shown in Figure 4. 

**Figure 5:**
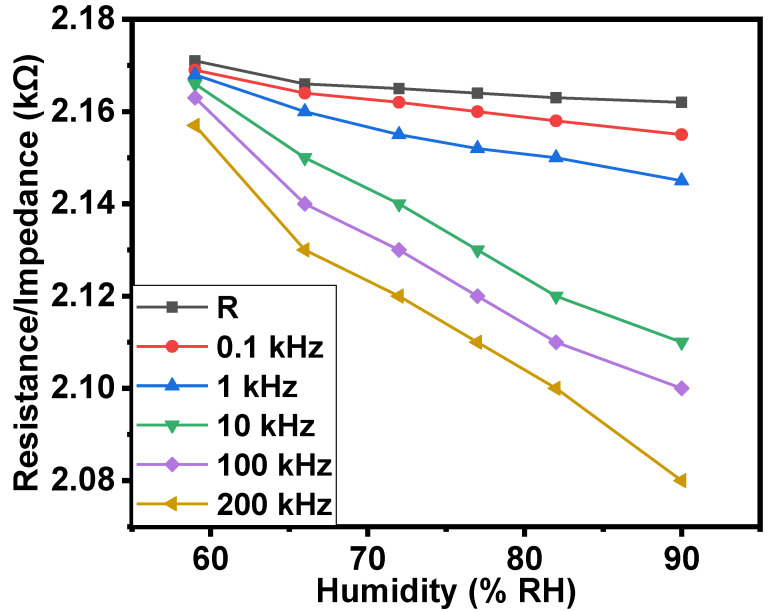
Resistance/impedance–humidity relationships of the surface-type shockproof resistor based on NiPc–oil jelly and CNT–oil jelly–rubber composite.

Similarly, when increasing the humidity from 59% to 90% RH (relative humidity), the resistance and impedance of the samples decreased by up to 1.04 times. The resistance/impedance–humidity relationships are shown in Figure 5. The R-squared (R^2^) values for the resistance–humidity and impedance–humidity relationships were also calculated. The value of R^2^ for the resistance–humidity relationship was 0.865, while for the impedance–humidity relationship, these values were in the range of 0.967 to 0.991.

Concerning the effect of humidity on the electric properties of the materials, two mechanisms may be considered: firstly, the diffusion of water molecules into material and increase in dielectric permittivity; secondly, the self-ionization of the water molecules into protons (H^+^) and hydroxide ions (OH) that, ultimately, leads to the separation of (H^+^) and (OH^−^), according to Equation (1), and the increase in the concentration of charges:

H_2_O <=> H^+^ + OH^−^ (1)

The mechanisms of the effect of humidity on the electric properties of the polymer materials were discussed in refs. [36,37]. The humidity sensors based on ceramic and polymers were also reviewed. Sensitivity, response time, stability and sensing mechanism were also discussed. The low dependence of the resistance and impedance of the investigated composite on the humidity may also be due to the presence of oil that retards the penetration of water molecules into the pores of the samples. In ref. [10], an organic positive (R-T)-characteristics resistor was fabricated. In ref. [6,11,12,13,14,38], details on different kinds of technologies, materials and properties concerning resistance were published, where the resistor fabrication technology was also developed. In particular, the composition and the process of producing electrical resistors were presented in refs. [6,11,12]. The method of manufacturing resistor paste was presented in ref. [13], while the composition and the properties of polymer thick-film resistors [14] and non-lead resistors [38] were also described. At the same time, it seems that organic materials applications in resistance technology can be realized and potentially used in practice. 

**Figure 6:**
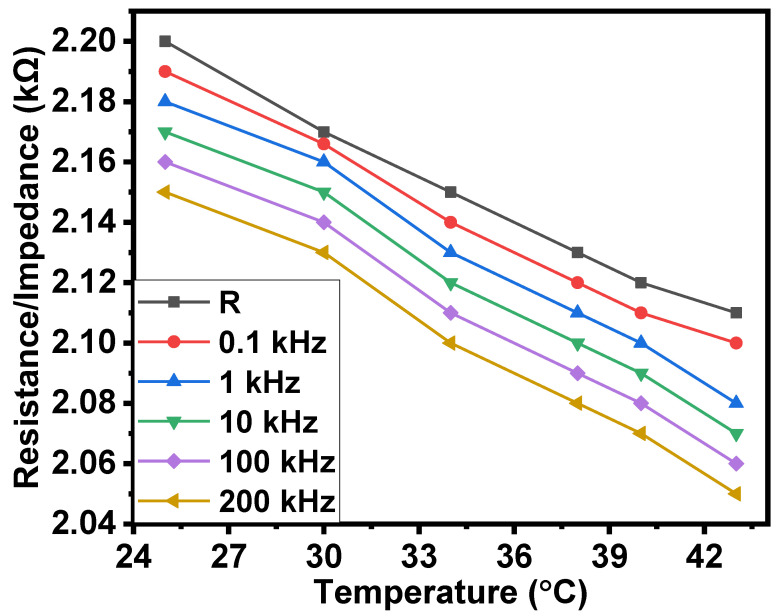
Resistance/impedance–temperature relationships of the surface-type shockproof resistor based on NiPc–oil jelly and CNT–oil jelly–rubber composites.

With the increasing temperature from 25 °C to 43 °C, the average decrease in the resistance and impedance was equal to 1.05, on average. The resistance/impedance–temperature relationships are shown in Figure 6.

The temperature coefficient of resistance (TCR) may be calculated as:

TCR = ΔR/RΔT(2)

where R is the initial resistance at temperature T_1_ and ΔR is the differences in resistance between temperatures T_1_ (initial temperature) and T_2_ (instantaneous temperature). The ∆T is the difference between initial and instantaneous temperatures. 

Similarly, the temperature coefficient of impedance (TCI) can also be introduced:

TCI = ΔZ/ZΔT(3)

where Z, ΔZ and ΔT are impedance, change in impedance at different temperatures and change in temperature ΔT.

Through these calculations, it was found that TCR was equal to −0.0023/°C. Using the same calculations, it was found that the temperature coefficient of impedance (TCI) was equal to −0.0023/°C at all frequencies in the interval of 0.1 kHz–200 kHz.

A comparison of the obtained values of TCR and the TCI with the TCRs of some metals that are used in electronics (such as silver (0.0038), copper (0.0039) and aluminum (0.0043)) shows that the resistance–temperature behavior of the NiPc–CNT–oil–rubber composite causes stress in metals. However, the resistance–temperature behavior of NiPc–CNT–oil–rubber composite is similar to semiconductors because, with the increasing temperature, the resistance/impedance decreases. In principle, this allows jelly-based and traditional metals-based composites to be fabricated with approximately zero temperature coefficients. 

**Figure 7:**
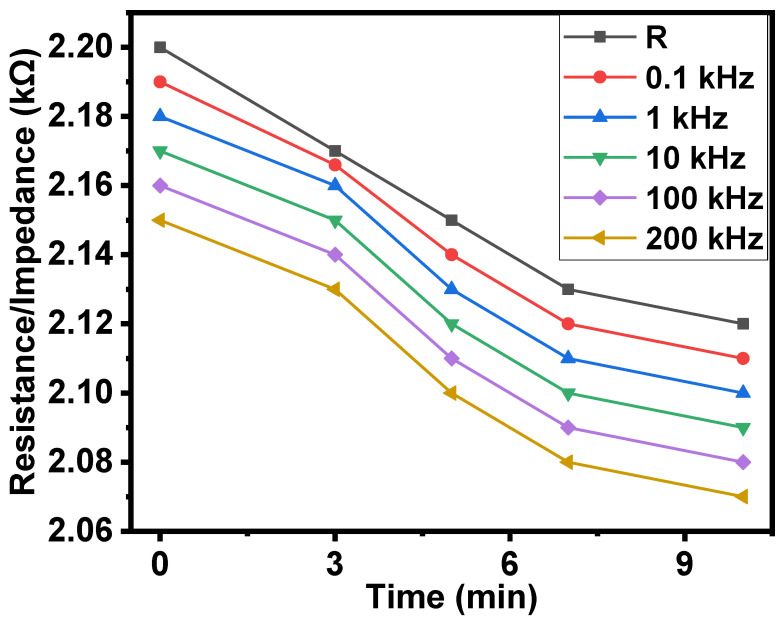
Effect of vibration on the resistance/impedance of the surface-type shockproof resistor with respect to time.

The effect of vibration on the resistance and the impedance of the samples is shown in Figure 7. Under the effect of vibration, the decrease in the resistance and impedance was observed as up to 1.03 times. 

The results are summarized in Table 1. It can be seen that the displacement, pressure, vibration, humidity and temperature have negligible effects on the fabricated resistor. 

The investigated samples can potentially be used as a prototype for the development of shockproof jelly electronic-based devices, particularly sensors. The impedance and resistance may also be changed if the ratio of the ingredients and type of ingredients are changed. A technological achievement regarding the fabrication of these devices is the use of edible organic oil, which could fabricate the uniform jelly type films of organic materials, which could not be realized simply by mixing “dry” ingredients. Especially, we would like to highlight that edible organic oil is environmentally friendly, unlike some other inorganic oils that are used in practice. This fact may be especially important in the implementation of the obtained results in practice.

The analysis of data received from the literature showed that the obtained results were supplementary. These results are primarily useful for the fabrication of cheap and flexible devices, which are especially important as a teaching aid. Secondly, these results support the investigation and understanding of the physical and electro-chemical properties of the flexible composites for their potential applications in the electronic devices which may be used in vibration conditions. Moreover, the jelly-based electronic devices are very attractive to be used successfully in vibration conditions.

## 3. Conclusions

The purpose of this research was the design, fabrication and investigation of the properties of shockproof, flexible, organic resistors. In this study, a rubber–jelly (NiPc–CNT-edible oil) composite-based flexible resistor was fabricated. The changes in the resistance and impedance of the resistor were measured under the effect of humidity, temperature, pressure, displacement and vibration. The results of this investigation showed that fabricated organic resistors are ecologically clean and environmentally friendly. This is due to the utilization of edible oil for the fabrication of the jelly that was used in the organic resistor: all organic components, including the organic semiconductor NiPc and carbon nanotubes were surrounded by edible oil. The presence of edible oil not only made the composite environmentally friendly but also provided an approximately constant resistance.

## 4. Experimental Section

**Figure 8:**
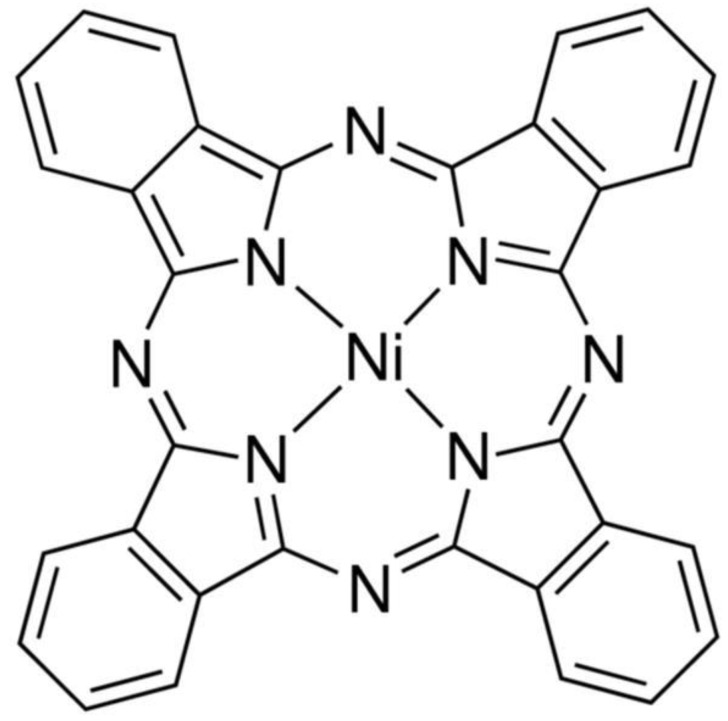
Molecular structures of NiPc.

For samples fabrication, we used edible oil (coconut oil and hempseed oil), organic semiconductor nickel phthalocyanine (NiPc), carbon nanotubes and rubber substrate. The CNTs (carbon nanotubes) and NiPc powder were purchased from Sun Nanotek Co., Ltd. and Sigma Aldrich, respectively. The diameter of the multi-walled carbon nanotubes was 10 to 30 nm, while their length ranged from 100 to 200 nm. The NiPc powder was used as it was received. Figure 8 shows the molecular structures of nickel phthalocyanine (NiPc). The molecular weight of the NiPc is 571.22.

**Figure 9:**
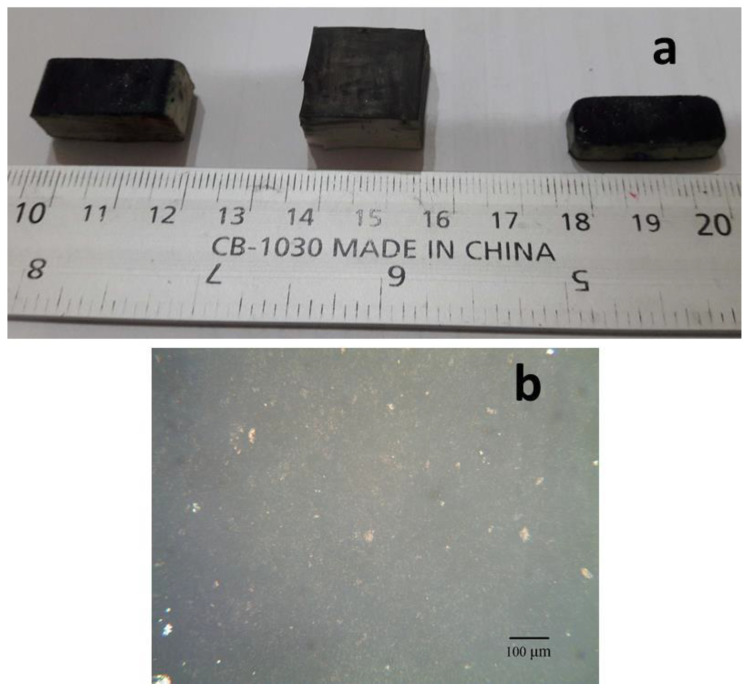
Pictures of the samples showing the 3D images of the rubber substrates covered with the CNT–oil and NiPc–oil jellies (**a**) and the magnified image of the top surface of the rubber substrate covered with the CNT–oil and NiPc–oil jellies (**b**).

The shockproof organic resistors, consisting of rubber–jelly (NiPc–CNT–oil) composites, were fabricated using rubbing-in technology. The effects of pressure, displacement, humidity, temperature and vibrations on the resistance and impedance of the resistors were studied. These jelly-based resistors were fabricated in the following way. The jelly of CNT and hempseed oil was prepared by mixing both 1:1 (50 wt.% and 50 wt.%). The jelly of NiPc and coconut oil was also prepared by mixing both of the ingredients in a ratio of 50 wt.% and 50 wt.%. The CNT–oil jelly was deposited on the rubber substrates by rubbing-in technology. The NiPc–oil jelly was deposited on the CNT–oil jelly layer. Figure 9a shows the 3D images of the samples, where the rubber substrates are covered with the CNTs-edible oil and NiPc-edible oil jellies. In these images, the front, top and side views of the rubber substrates can be clearly seen. Figure 9b shows the magnified image of the top surface of the rubber substrate covered with the CNTs-edible oil and NiPc-edible oil jellies.

**Figure 10:**
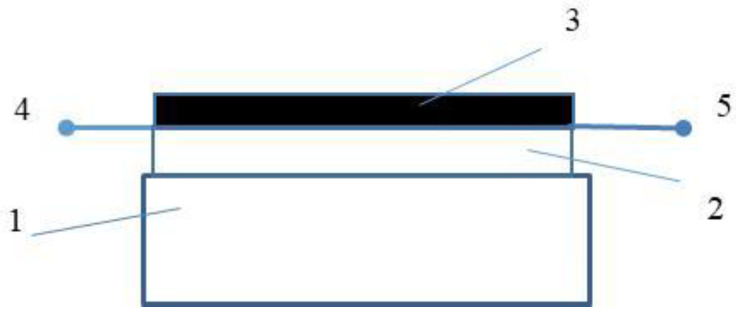
Schematic diagram of the surface-type shockproof NiPc–CNT–oil–rubber composite sample: rubber substrate (1), CNT–oil jelly (2), NiPc–oil jelly (3), terminals (4 and 5).

Figure 10 shows the schematic diagrams of the surface type resistors, which were fabricated using rubbing-in technology. The total thickness of the jelly films ranged from 20–26 µm. The size of the surface-type resistors was equal to 2:0.7:0.7 cm^3^. Displacement and pressure were applied along the length of the samples. For the measurements of the impedance in the range of frequencies from 100 Hz to 200 kHz, the digital LCR meter MT-4090 was used. The temperature was measured using a Fluke-87 multimeter, while the humidity was measured using Fisher scientific humidity meter.

## Figures and Tables

**Table 1:** Summary of the results showing the effect of various parameters on the resistor.

Parameter	Range	∆R (Times)	∆Z at 100 Hz (Times)	∆Z at 1 kHz (Times)	∆Z at 10 kHz (Times)	∆Z at 100 kHz (Times)	∆Z at 200 kHz (Times)
Displacement	0–50 μm	−1.025	−1.028	−1.032	−1.037	−1.039	−1.040
Pressure	0–850 gf/cm^2^	−1.081	−1.080	−1.080	−1.081	−1.080	−1.078
Humidity	60–90%RH	−1.004	−1.006	−1.01	−1.03	−1.03	−1.04
Temperature	25–43 °C	−1.042	−1.042	−1.048	−1.048	−1.048	−1.048
Vibration	0–10 min	−1.037	−1.038	−1.038	−1.038	−1.038	−1.038
